# Infantile haemangiomas do not occur more frequently in children with congenital melanocytic naevi

**DOI:** 10.1111/bjd.14791

**Published:** 2016-12-07

**Authors:** V. Martins da Silva, V. Kinsler

**Affiliations:** ^1^ Genetics and Genomic MedicineUCL Institute of Child HealthLondonU.K.; ^2^ Department of MedicineUniversity of BarcelonaBarcelonaSpain; ^3^ Department of Paediatric DermatologyGreat Ormond Street Hospital for ChildrenLondonU.K.

dear editor, Infantile haemangioma (IH) is a very common benign vascular tumour with a reported incidence of 4–10% in infants,^1^ and no clear genetic basis described as yet.^2^ Congenital melanocytic naevi (CMN) are benign melanocytic tumours present in 1% of newborns, which when multiple are caused by post‐zygotic mutations in the gene *NRAS* in the majority of cases,^3^ and when single, carry various somatic mutations where causality is difficult to prove.^4^, ^5^, ^6^ Melanocytic and vascular anomalies can coexist in the condition phakomatosis pigmentovascularis (PPV), and the same genetic mutation is responsible for both cutaneous lesions;^7^ however, these do not involve either CMN or IH. Moreover, the vascular lesion in PPV is considered congenital and malformative as CMN, and not proliferative and acquired as IH.^8^ A case series of six patients presenting with both CMN and IH has been reported previously, where the authors hypothesized that this co‐occurrence might be more common than expected by chance.^9^

To test this hypothesis we conducted a systematic evaluation of the presence of IH in the cohort of patients with CMN seen in our tertiary referral service over a 10‐year period between March 2006 and February 2016. All children were examined by the same physician, and data were collected prospectively. We included in this analysis only children less than 3 years of age at the examination date, as the natural history of IH is to spontaneously involute during the first few years of life.

A total of 244 patients with CMN under the age of 3 years were seen in this time period, with a mean and median age of 0·78 years and 0·53 years, respectively. Of these, 142 were females, giving the same male : female ratio of 1 : 1·4 as has previously been reported for our CMN cohort.^10^ Fourteen patients were recorded as having an IH (5·7%), compatible with prevalence figures for the general population. Furthermore, the characteristics of those with an IH mirror those of the general population, as the male : female ratio for those with IH and CMN was 1 : 6. Table 1 shows the clinical characteristics of the patient cohort, comparing those with and without IH. The number of patients with CMN and IH is too small to perform a statistical comparison of the severity of CMN phenotype, but clinical phenotyping data are shown in Table 1.

**Table 1:** Clinical characteristics of patients with congenital melanocytic naevi (CMN) with and without infantile haemangioma (IH)

	Patients with CMN, *n* (%)	Patients with CMN + IH, *n* (%)
Sex		
Female	130 (56·5)	12 (85·7)
Male	100 (43·5)	2 (14·3)
Total	230 (100)	14 (100)
Projected adult size		
< 10 cm	58 (25·2)	1 (7·1)
10–20 cm	45 (19·6)	3 (21·4)
20–40 cm	52 (22·6)	4 (28·6)
40–60 cm	25 (10·9)	3 (21·4)
> 60 cm	39 (17)	1 (7·1)
Multiple small or medium	7 (3)	2 (14·3)
Missing	4 (1·7)	0
Approximate total number of naevi at examination date		
1	34 (14·8)	0
2–9	55 (23·9)	2 (14·3)
10–19	33 (14·3)	2 (14·3)
20–50	33 (14·3)	0
50–100	25 (10·9)	0
100–200	17 (7·4)	2 (14·3)
> 200	4 (1·7)	1 (7·1)
Missing	29 (12·6)	7 (50)
Location of principal CMN		
Face	17 (7·4)	0
Scalp	21 (9·1)	1 (7·1)
Neck	1 (0·4)	0
Trunk	80 (34·8)	3 (21·4)
Limb	26 (11·3)	3 (21·4)
Scalp, neck and trunk	8 (3·5)	0
Face and scalp	16 (7·0)	0
Multiple	4 (1·7)	1 (7·1)
Missing	57 (24·8)	6 (42·9)
Location of haemangioma		
Face		1 (7·1)
Head and neck (nonfacial)		0
Trunk		6 (42·9)
Extremity		3 (21·4)
Missing		4 (28·6)

This systematic study of the prevalence of IH in a cohort of patients with CMN has found no increase above that of the normal population, and a sex ratio in line with what we would expect for IH alone. This study does not support a connection at a genetic level between CMN and IH, either at germline predisposition or at somatic mutation level.
